# Effect of electrochemical oxidation and drug loading on the antibacterial properties and cell biocompatibility of titanium substrates

**DOI:** 10.1038/s41598-022-12332-z

**Published:** 2022-05-21

**Authors:** Fateme Nowruzi, Rana Imani, Shahab Faghihi

**Affiliations:** 1 Department of Biomedical Engineering, Amirkabir University of Technology, Tehran, 15875/4413 Iran; 2 Stem Cell and Regenerative Medicine Group, National Institute of Genetic Engineering and Biotechnology (NIGEB), Tehran, 14965/161 Iran

**Keywords:** Biotechnology, Biomaterials, Nanobiotechnology

## Abstract

A combination of ${\text{ TiO}}_{2}$ nanotube array (TON) and controlled drug release system is employed to provide enhanced surface properties of titanium implants. Electrochemical anodization process is used to generate TON for introducing, vancomycin, an effective antibacterial drug against *Staphylococcus* *aureus*. TON loaded vancomycin is then coated with a number of layers of 10% gelatin using spin coating technique. The gelatin film is reinforced with graphene oxide (GO) nanoparticles to improve the surface bioactivity. The surface of the samples is characterized by field emission electron microscopy (FESEM), energy-dispersive X-ray spectroscopy (EDS), and contact angle measurement. The results illustrate that the TON was constructed and vancomycin molecules are successfully loaded. The drug release study shows that the amount of released vancomycin is controlled by the thickness of gelatin layers. With an increase in gelatin film layers from 3 to 7, the release of vancomycin in the burst release phase decreased from 58 to 31%, and sustained release extended from 10 to 17 days. The addition of GO nanoparticles seems to reduce drug release in from 31 to 22% (burst release phase) and prolonged drug release (from 17 to 19 days). MTT assay indicates that samples show no cytotoxicity, and combination of GO nanoparticles with gelatin coating could highly promote MG63 cell proliferation. Soaking the samples in SBF solution after 3 and 7 days demonstrates that hydroxy apatite crystals were deposited on the TON surface with GO-gelatin coating more than surface of TON with gelatin. Moreover, based on the results of disc diffusion assay, both samples (loaded with Vancomycin and coated with gelatin and gelatin-GO) with the inhibition zones equaled to 20 mm show effective antibacterial properties against *S*. *aureus*. The evidence demonstrates that titania nanotube loaded with vancomycin and coated with gelatin-GO has a great potential for general applicability to the orthopedic implant field.

## Introduction

Titanium and its alloys are among the most widely used metallic materials in the field of orthopedic and dental implants^1–3^. Desirable properties, including tensile strength and elastic modulus, high biocompatibility, and low risk of allergy make them successful candidate for these applications^4–6^. However, Ti-based implants some deficiencies including poor osteointegration, low osteogenesis, and bacterial infection in the implant site which may lead to implants failure especially in prolonged use^7–9^. Generally, poor osteointegration of Ti-based implants occurs because of bio-inert surface, production of excessive reactive oxygen species (ROS) at the interface, and bacterial infection after surgery^10^. As a result, two strategies are commonly considered to moderate these problems including increasing osteoblast cell proliferation and inhibition of bacterial infection^11,12^.

Long-term intravenous or oral antibiotics are the main traditional approaches to treat bacterial infection after implant surgery^13^, which have severe drawbacks including harmful side effects, toxicity, uneven bio-distribution, and less bioavailability^14,15^. *Staphylococcus aureus* is considered the main bacteria that causes implant infection after surgery^16–18^. The secretion of these bacteria on implant surfaces forms biofilms, which protect them against the immune system and antibacterial agents. Consequently, treatment with antibiotics has become a vital issue^19^. Since bacteria and osteoblast cells are in competition for attachment, implants having antibacterial surface characteristics can decrease bacterial attachment and colony formation so they can be used for treating bone defects^20,21^. Advanced biomaterials equipped with sustained and localized release of antibacterial agents could promote the healing/regeneration process of tissues which are more susceptible to bacterial infections (e.g. bone, skin, cardiac tissue)^22–25^.

With the convergence of material science and biology, a combination of surface modification and drug delivery systems can be employed to tackle the above mentioned complications. The electrochemical anodizing is a prevalent process among surface modifications that constructs ${\text{TiO}}_{2}$ nanotube arrays (TON) on titanium substrates^26–29^. TON arrays have beneficial properties including hosting a vast range of drugs and higher biocompatibility due to their highly porous nanostructures. Moreover, their fabrication is low cost and simple^29–33^. The main disadvantage of TON arrays as drug nano-reservoirs is uncontrollable drug release behavior that may cause toxicity in the implant site. However, modified TON, such as TON coated with biopolymers, exhibits enhanced drug release behavior^34–36^. TON coated by biopolymers can provide more controllability on drug release behavior by selecting a biopolymer based on composition and degradation rate properties^37^. In addition, the polymer coating can inherently have osteointegration and antibacterial properties^38–40^, and they can also carry a second drug as a multidrug system^41–45^.

In this study, in order to delay the drug release while enhancing the bioactivity of titanium substrates, a multilayer nanocomposite hydrogel was used as a cover on the TON surface. Among the biocompatible hydrogels, gelatin with FDA approval serves as the most important biopolymer which frequently used in biomedical applications, especially in tissue engineering and drug delivery researches^46^. According to literatures and our previous experience, GO, as hydrophilic nanoparticle provides many advantages in combination with hydrogels as a nanocomposite structure^47,48^. In dental and bone tissue engineering, GO nanoparticles enhance osseointegration/osteoblast bioactivity via acceleration of apatite formation^49^. In addition, graphene and its derivatives show great potential for osteogenic differentiation of stem cells^50^ and have been suggested as a coating agent on the orthopedic implants^51,52^. Here, we aimed to superpose gelatin and GO properties in order to modify TON surface where the coated gelatin/GO multilayer structure improves osteogenic properties at the same time as delaying drug release from nanotube reservoir.

For this purpose, TON arrays were formed by anodizing process on the surface of titanium substrates and Vancomycin, an antibacterial drug against *S. aureus* was introduced into nanotube arrays^53,54^. Graphene oxide-gelatin nano-composite was used to coat TON arrays loaded with Vancomycin. The modified titanium samples then were characterized by field emission scanning electron microscopy (FESEM), energy-dispersive X-ray spectroscopy (EDX), wettability measurement, and degradability. In addition, the drug release behavior was assessed on samples having different layers of coating. The effect of surface modification of titanium samples was assessed on the growth of MG63 cells and formation of hydroxyapatite crystals by MTT assay and bioactivity test. Finally, disc diffusion assay was employed to elucidate antibacterial activity of the samples.

## Experimental

### Materials

Commercially available pure titanium (CP-Ti-grade 2) used in this study were supplied by McMaster Carr Company, Los Angeles, CA, USA. Graphene oxide was obtained from Central Laboratory of Amirkabir University, Iran, Tehran. Vancomycin Hydrochloride was purchased from Daana pharmaceutical company, Iran, Tehran. Dulbecco’s modified eagle medium (DMEM) and trypsin were purchased from Gibco BRL (France). Fetal bovine serum (FBS), MTT, DMSO, PBS, and penicillin/streptomycin (PS) were purchased from Sigma-Aldrich. Simulated body fluid (SBF) was provided by APATECH, Iran, Yazd. Other chemicals were supplied by Merck, Germany.

### Preparation of ${\text{TiO}}_{2}$ nanotube arrays (TON)

Highly-ordered titania nanotube arrays were fabricated on titanium discs of 10 mm diameter using electrochemical anodization method. The samples were first grounded with SiC abrasive paper (grit size: P800, P1000, P1200, and P1500) and sequentially cleaned in ethanol 70%, acetone, and deionized water using an ultrasonic bath for 25 min. The samples were dried at ambient temperature before anodization. For anodization process, stainless steel and titanium discs having smooth surfaces were used as cathode and anode electrodes, respectively. The cell was connected to DC power supply (PEQ lab, EV843, made in Belgium and ethylene glycol, 38 wt% ammonium fluoride, and 2 vol% deionized water was used as electrolyte solution. Processing parameters including temperature, voltage, and time have significant effects on the structure of anodized titanium. The anodization process was carried out at voltages of 45, 60, 70, and 95, for 1.5 and 3 h (Table 1), at a controlled temperature (4 °C). All anodized samples were ultrasonically cleaned in ethanol and acetone for a minute and rinsed with deionized water. Finally, samples were dried at room temperature.

**Table 1:** Time and voltage used in anodizing process.

Voltage (V)	Time (h)
45	1.5
45	3
60	1.5
60	3
70	1.5
70	3
90	3

### Vancomycin loaded into TON arrays on Ti surface

The Vancomycin was introduced into titania nanotubes according to a method of pipetting and drying. 25 mg/ml solution of Vancomycin in phosphate buffer saline (PBS) was prepared and 10 µL of drug solution was pipetted onto nanotubes. The samples were dried at air temperature and cleaned with soft tissue. Finally, to remove the drug residues, the samples were rinsed with the PBS solution. To enhance the drug loading efficiency and achieve the required concentration, the drug loading process was repeated four times.

### Coating of gelatin-based film on TON arrays

Gelatin-based films with different thicknesses were coated on titania nanotube substrates using spin-coating method. A 10 wt% gelatin aqueous solution was prepared and spin-coated on the substrates at a speed of 3800 RPM for 60 s. The polymer film thickness would affect sustained release behavior of the drug, therefore, samples with varied coating layers were prepared to obtain desired drug release profile. To modify gelatin film, GO was added as an agent. For GO/gelatin composite synthesis, GO nano-powders were first dispersed in deionized water using an ultrasonic homogenizer for 30 min. The gelatin10%-GO0.5% solution was then prepared and spin-coated on the samples. All the samples were subsequently glutaraldehyde vapor treated with glutaraldehyde 25%, at 25 °C. To remove excess glutaraldehyde, samples were then immersed in a glycine aqueous solution (7.5 mg/mL) for 5 min and dried at room temperature. Finally, for sterilization of the samples Ultraviolet radiation was used for 20 min. Table 2 lists the samples with their abbreviations.

**Table 2:** Samples and related abbreviations.

Samples	Abbreviations
${\text{TiO}}_{2 } \;{\text{nanotube}}\;{\text{arrays}}$ (Titania nanotube)	TON
${\text{TiO}}_{2 } \;{\text{nanotubes}}$ loaded with Vancomycin	TON-Van
${\text{TiO}}_{2 } \;{\text{nanotubes}}$ loaded with Vancomycin and coated with Gelatin 10% (3 layers)	TON-Van-Gel C3
${\text{TiO}}_{2 } \;{\text{nanotubes}}$ loaded with Vancomycin and coated with Gelatin 10% (7 layers)	TON-Van-Gel C7
${\text{TiO}}_{2 } \;{\text{nanotubes}}$ loaded with Vancomycin and coated with gelatin10%-GO0.5% (7 layers)	TON-Van-Gel-GO

### Surface characterization

Field emission scanning electron microscopy (FESEM) was utilized to observe the morphology of anodized samples as well as gelatin-based coated ones. After the samples were coated with an ultrathin gold layer via sputter coater (Emitech, K450X), images were captured at an accelerating voltage of 25 kV in high vacuum mode. The titania nanotube dimensions were analyzed using the Digimizer software. The non-destructive ellipsometry (SENpro, SENtech, Germany) test and SEM analysis were performed to measure the thickness of polymer coating. Energy-dispersive X-ray spectroscopy (EDS) was used to determine the types of elements in the samples (TON and TON-Van), especially to verify the presence of Vancomycin.

### Wettability measurement

The sessile water drop technique was used to measure the static contact angle and wettability of the samples. Deionized water droplet (0.5 µL) was firstly deposited on the top surface of samples through micro-syringe. Images were then captured and the static contact angle was measured using CAG-20, Jikan Co. The experiment measured the average contact angle at five different points in each sample at ambient temperature.

### Degradability evaluation

Degradability of coating has a direct effect on drug release behavior of the samples. The degradation of biopolymers is related to their composition, structure, charge, and surface properties. In general, weight loss measurement is the most common method to investigate hydrogel degradation. However, in this study, weight loss measurement was not applied because there was a significant difference between coating weight and titanium weight. Therefore, degradability was evaluated by comparing the SEM images of coating before and after drug release in PBS solution. Samples were placed in clean and sterile glass bottles containing PBS. Then they were sealed and placed in an incubator (GFL, Germany) at 37 °C, at a speed of 60 RPM. The degradation was measured after 10 and 17 days. Eventually, freeze-drying was performed to remove the water that was absorbed by the hydrogel coatings.

### Vancomycin release

In-vitro release of Vancomycin from bare TON, Gelatin coated, and gelatin-GO coated samples loaded with the drug were investigated by immersion into 10 mL of PBS (pH = 7.4) at 37 °C. During the first 6 h, 300 µL of the medium was taken out at short intervals to monitor the burst release of the drug. For delayed release of the drug, 300 µL PBS solution was collected every 24 h until the whole drug was released into the medium. The calibration curve was plotted based on the Vancomycin in PBS, the release of the drug was measured by UV-spectroscopy (Lambda 900, PerkinElmer, USA) at 280 nm. The release was considered complete when there was no change in the absorbance at 280 nm. The data were analyzed using mathematical kinetic modeling.

### Cell culture

The osteosarcoma cell line Human Osteoblast-Like cells (HOS) MG-63, from the National Cell Bank of Iran (NCBI; Pasteur Institute), was used for cell culture experiments. The cells were cultured in a culture medium (DMEM supplemented with 10% FBS, 1% penicillin/streptomycin) at 37 °C in the humidified incubator with 5% CO_2_. After the culture reached approximately 90% confluence, MG-63 cells were removed from culture flasks by trypsinization and centrifuging (1500 RPM, 5 min). They were then suspended in the fresh medium.

### Cell viability

Samples including TON, TON-Van-Gel C7, TON-Van-Gel-GO and culture plate as a control were sterilized by UV and washed with PBS and culture medium before they were placed in a 24-well plate. Then, 200 µL of culture medium containing 80,000 cells were seeded onto the surface of the samples. After incubation for 3 h, 1 mL of culture medium was added to each well. Subsequently, after 1, 3, and 7 days of incubation, to remove the unattached cells, the culture medium was removed and samples were rinsed with sterile PBS twice and incubated with fresh culture medium supplemented with 1 mg/mL MTT solution for 4 h to allow formazan formation. After removing the medium, 50 µL of dimethyl sulfoxide (DMSO) solution was added to each well and incubated for 10 min to dissolve the formazan crystals. The medium was transferred to a 96-well plate to measure the absorbance of the resulting purple solution using a micro-plate reader at 580 nm. The cell viability directly depends on the amount of formazan crystals, calculated by dividing the average optical density of samples by the average optical density of control.

### Bioactivity

Samples (TON-Van-Gel and TON-Van-Gel-Go) were evaluated by soaking in 5 mL of SBF solution similar to human blood plasma. The samples were kept at 37 °C for 3 and 7 days. The medium was replaced every 2 days to avoid insufficient presence of ions (Ca and P). The samples were removed from the SBF solution and dried using freeze-drying. The structure and morphology of the SBF-immersed samples were characterized by FESEM.

### Antibacterial assay

A zone of inhibition test, also called a Kirby-Bauer Test, was utilized to investigate the antibacterial characteristics of the samples. Gram-positive *S. aureus* (ATCC 25923), one of the most infectious bacteria in bone tissue engineering was employed. Plates containing Mueller–Hinton agar medium (Merck, Germany) were spread with *S. aureus* at a concentration of 1.5 × 10^8^ CFU/mL. Then, the samples (TON-Van-Gel-GO and TON-Van-Gel C7) were placed gently into the agar nutrient. Ciprofloxacin antibiotic discs were placed on the plates as a positive control. Finally, the plates were incubated for 24 h at 37 °C in order for bacteria to grow in agar media. The size of the inhibition zone that appeared around the discs indicates the antibacterial activity of each sample.

### Statistical analysis

Statistical analysis was carried out using SPSS software (v 17.0; IBM New York, NY, USA) when statistical differences were detected, a t-student comparison test was performed. Data are reported as mean ± SD at a significance level of *p* < 0.05.

## Results and discussion

### Fabrication and characterization of titania nanotube arrays

**Figure 1:**
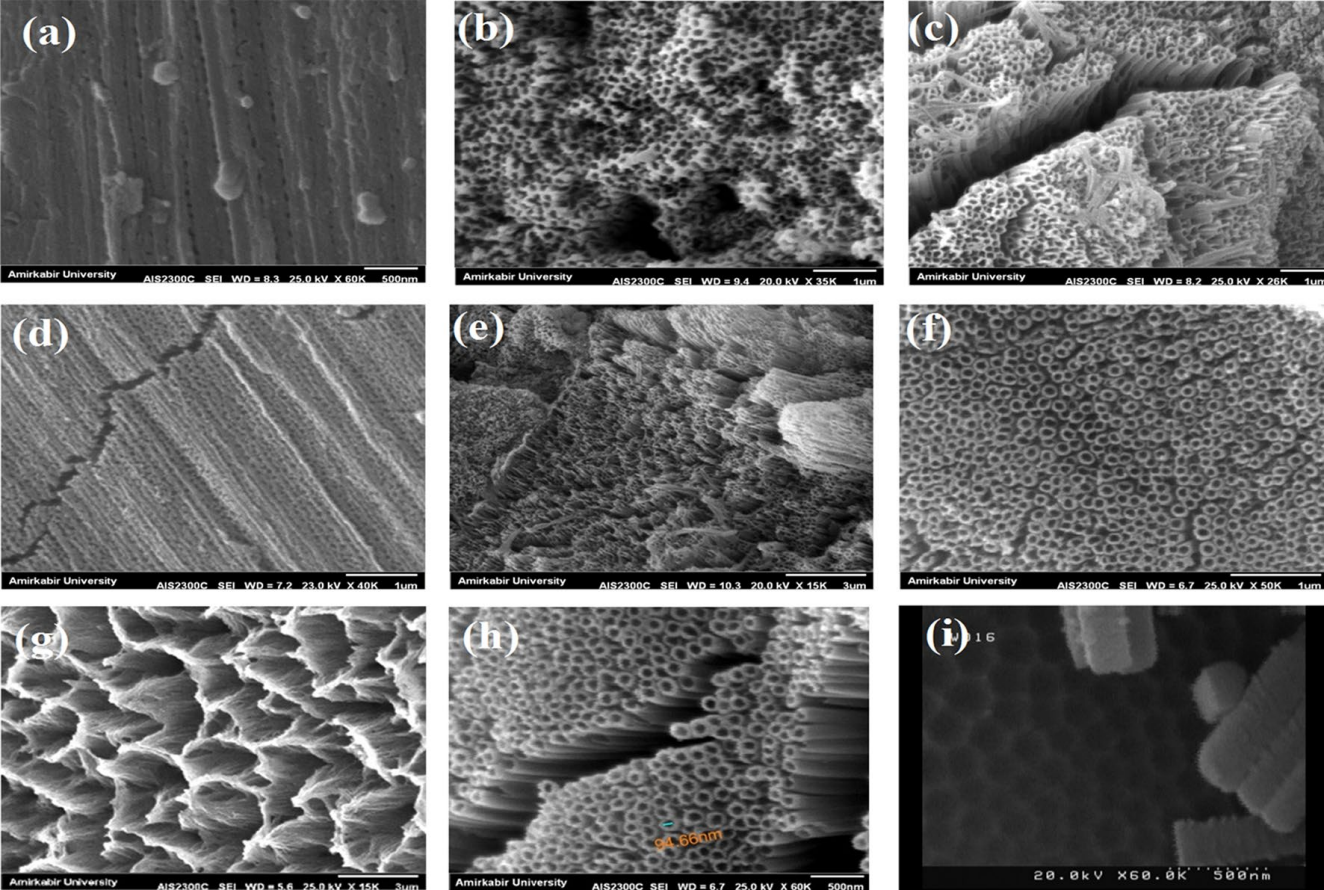
SEM images of samples after anodizing process with different voltages and times **(a)** 45 V for 1.5 h, **(b)** 60 V for 1.5 h, **(c)** 70 V for 1.5 h, **(d)** 45 V for 3 h, **(e)** 60 V for 3 h, **(f)** 70 V for 3 h, **(g)** 90 V for 3, **(h)** opened top and **(i)** closed bottom of TON array constructed by anodizing process with voltage of 70 V and 3 h.

Recently, more attention has been focused on the electrochemical anodization to construct self-order titania nanotube structures. Strong adherent nanotube structures with open tops and closed ends are the most favorable drug nano-carrier systems. The parameters such as voltage and time of the anodization process have the main impact on the morphology of titanium dioxide. Here, the impact of anodizing parameters on the morphology of titania, which has great importance for the consequent drug loading was investigated. The anodizing process with different voltages (45, 60, 70, 90 V) and various times (1.5, 3 h) was carried out. The experiments were performed in the electrolyte containing ammonium fluoride at the controlled temperature (4 °C). The first reaction of anodizing oxidation was the electrolysis of water (reaction 1). A compact layer of titanium dioxide was then formed on the titanium substrates (reaction 2) which was dissolved by fluoride ions, subsequently. As the results, the pits were formed on the titanium surfaces (reaction 3). At the suitable voltage and time, pits were converted into nanotube structures. At the lowest applied voltage (45 V), the pits with infinitesimally small diameters were formed on the Ti surfaces, however, the nanotube morphology was not apparent (Fig. 1a). When the voltage was increased to 60 V, the titania nanotubes were formed, yet they were not highly ordered (Fig. 1b). At the voltage of 70 V, the nanotubes showed a more distinguishable morphology with increased diameter (Fig. 1c).

1 $$ 2{\text{H}}_{2} {\text{O }} \leftrightarrow {\text{O}}_{2} + 4{\text{H}}^{ + } + 4{\text{e}} $$

2 $$ {\text{Ti}} + {\text{ O}}_{2} { } \leftrightarrow {\text{ TiO}}_{2} $$

3 $$ {\text{TiO}}_{2} + 6{\text{F}}^{ - } + { }4{\text{H}}^{ + } \leftrightarrow { }[{\text{TiF}}_{6} ]^{2 - } { } + { }2{\text{H}}_{2} {\text{O }} $$

With increasing time of anodizing from 1.5 to 3 h, no significant changes were observed in the nanotube structure at the low voltage (45 V) (Fig. 1d). In the higher voltage though, extending the anodizing time made the nanotubes provided a more intrinsic structures (Fig. 1e,f). Increasing the voltage to 90 V was gradually destroyed the top portion of the nanotubes (Fig. 1g).

A more desirable morphology of nanotubes was obtained at the anodizing voltage of 70 for 3 h, where the SEM images revealed that they possess a diameter of about 94 ± 4 nm and length of about 3 µm, aligned vertically and highly-ordered with opened top and closed bottom (Fig. 1h,i).

### EDS analysis of TON and TON-Van

**Figure 2:**
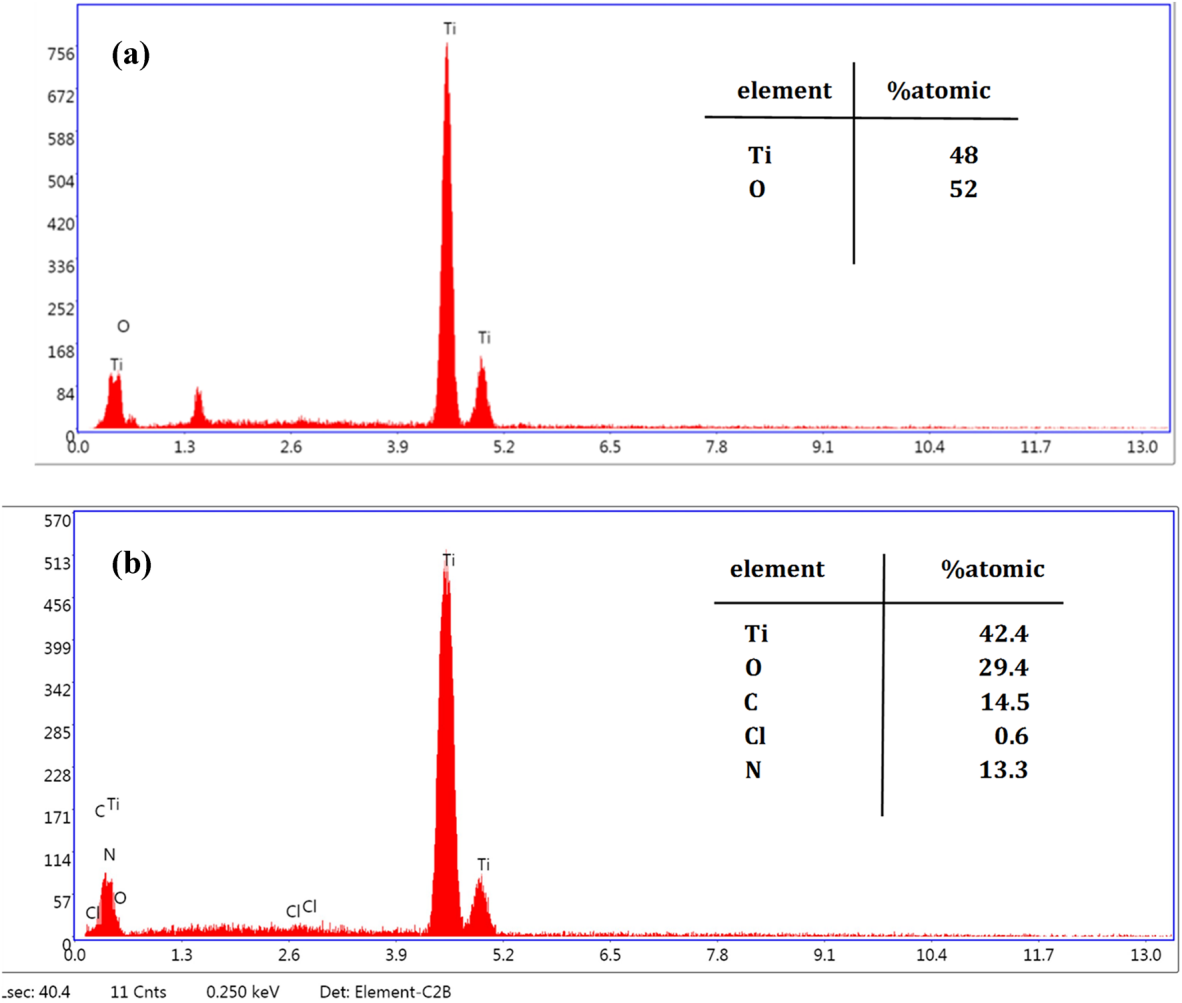
EDS spectrum of **(a)** TON, **(b**) TON-Van.

EDS was utilized to prove that Vancomycin was loaded into titania nanotube arrays. Chemical compositions of samples (TON and TON-Van) were detected, as illustrated by Fig. 2a,b. Ti and O elements could be seen in both samples, confirming ${\text{TiO}}_{2}$ formation on the surface of titanium samples. The presence of Cl, N, and C in the EDS spectrum of TON-Van (Fig. 2a) indicates that Vancomycin (C66H75Cl2N9O24) has been successfully loaded into the nanotube arrays.

### Current transient curve and anodizing process evaluation

**Figure 3:**
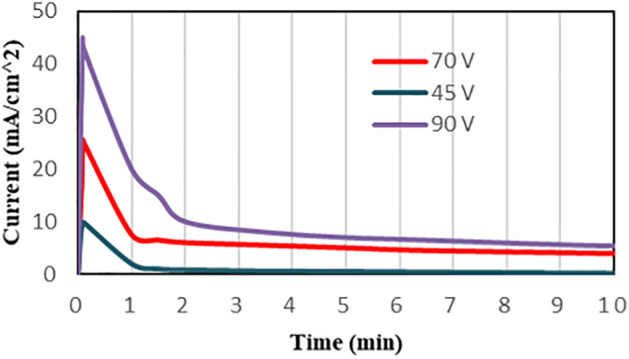
Transient curve at voltages of 45, 70, and 90 V for 3 h (4 °C).

Current transient curves demonstrate the behavior of current versus time during the anodizing process. This curve can help to explain the formation of titania structure during the process. Figure 3 displays the current transient curve at the voltage of 70, for 3 h, at the temperature of 4 °C. The application of initial voltage showed a sharp rise up to a which is attributed to the electrolysis of water and the beginning of the formation of the compact oxide layer. The observed gas evolution in the anode cell seems to be related to the transfer of electric charge and indicates the electrical conductivity of the titanium surfaces. Subsequently, the current declines sharply due to the formation of a dense and compact oxide layer with a low electrical conductivity. As the transfer of electric charge decreased, ion transportation in the electrolyte increased. At the next step the compact layer was dissolved by fluoride ions and pores in the dense layer were nucleated which causes a slight increase in the current, followed by a dropdown. However, the small increase in current was not observed here due to the fast ion transportation (especially fluoride ions) and rapid dissolution of the oxide layer. The other reason could be the limitation on the time resolution of our measurement device to record the rapid changes in the current. The decrease in current would gradually turn to a plateau. There was a competition between oxidation and dissolution of the oxide layer which controls the anodizing process. Nanotubes were formed on the compact oxide layer as the anodization proceeds. As can be seen in Fig. 3, current increases to higher values with the increase of voltage. This is due to the increase of electron exchange during the anodization process. As the voltage of the anodization process increases, the oxidation rate improves and the exchange of electrons and current density enhances.

### Water contact angle measurement

**Figure 4:**
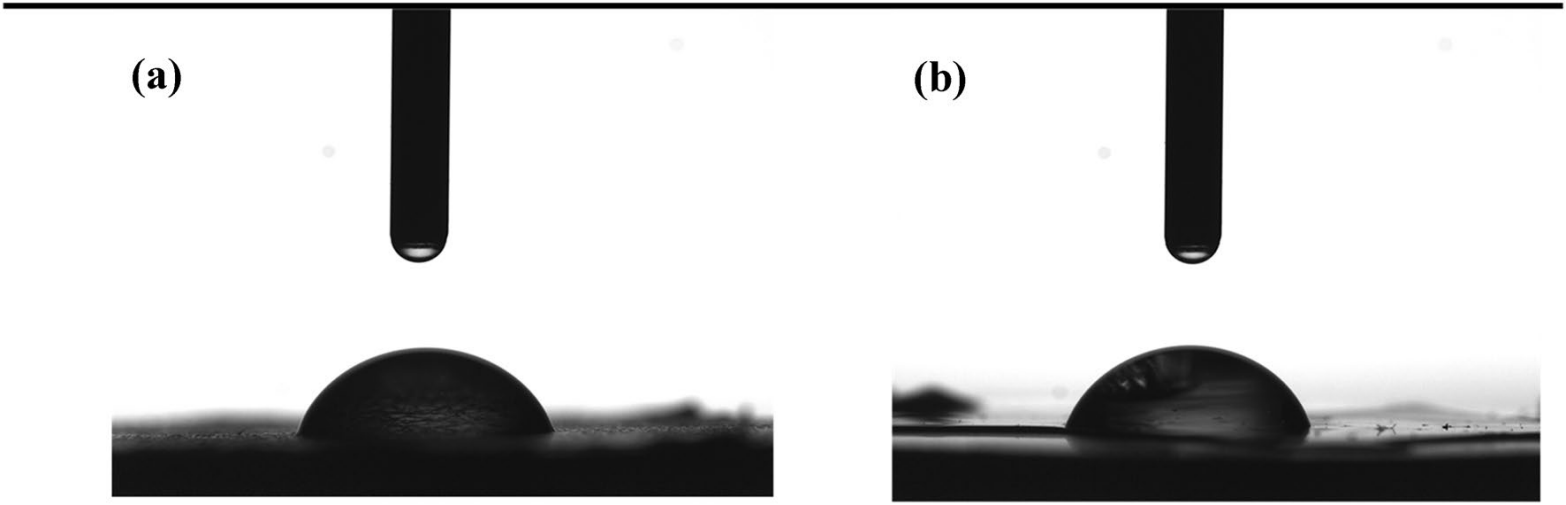
Water contact angle measurements of **(a)** TON-Gel, **(b)** TON-Gel-GO.

Surface hydrophilicity or wettability of implants has a significant impact on cell behavior. Moreover, the water-soluble drug loading efficiency also improves with increasing wettability. Therefore, contact angle measurement, the primary way to investigate the degree of affinity between water and the surfaces, becomes vital to characterize the surfaces of drug delivery implants. Here, the wettability of samples (TON, TON-Gel C7, and TON-Gel-GO) was studied and depicted in Table 3. The TON was hydrophilic due to water penetration into the nanotubes. The contact angle of TON-Van-Gel C7 and TON-Gel-Go were 71.9° and 70.2°, respectively, which are in a similar hydrophilic range (smaller than 90°) (Fig. 4a,b). The similar contact angle indicates that even by modifying gelatin with graphene oxide as a hydrophilic agent, the contact angle was unchanged. This could be partly due to graphene oxide nanoparticles which fill and block the micro-pores among the gelatin molecules. The micro-pores structure provides the channels for penetration and absorption of water molecules. This could also be related to the strong crosslink between the chains of polymer which prevents the water absorption.

**Table 3:** Water contact angle measurements of samples.

Samples	Contact angle (degree)
TON	< 5°
TON-Van-Gel C7	71.9 ± 1.34
TON-Van-Gel-GO	70.2 ± 0.28

### In-vitro degradation of gelatin-coating

**Figure 5:**
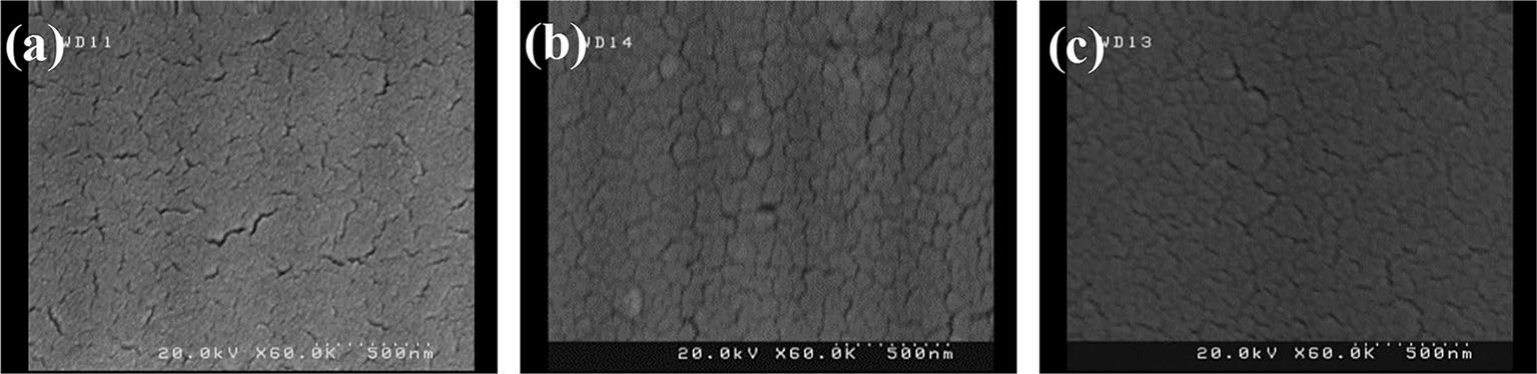
SEM images of TON-Van-Gel C7 soaked in PBS and freeze dried **(a)** before drug release **(b)** after 10 days, and **(c)** after 17 days.

The degradation of coating is an important factor for drug release behavior, therefore, the in-vitro degradation of the TON-Van-Gel C7 was evaluated. FESEM was utilized to observe the changes in the microscopic morphology of gelatin coating during the degradation process in PBS. Figure 5a reveals the dense structure of gelatin coating with some cracks before degradation. These cracks are probably due to the process of freeze-drying. Figure 5b,c show no holes and collapse in the surface coatings after 10 and 17 days which implies the dense structure of the coating possibly due to the strong crosslink between the chains of polymer.

### Vancomycin release

**Figure 6:**
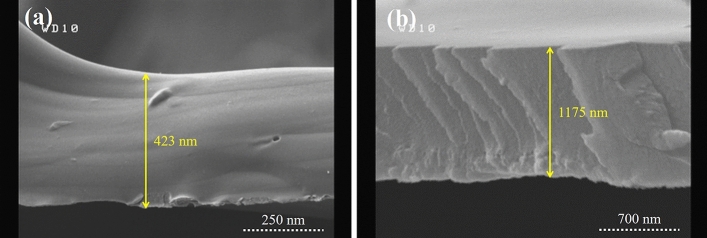
Cross sectional SEM images of Gelatin-coating **(a)** 3 layers and **(b)** 7 layers.

**Figure 7:**
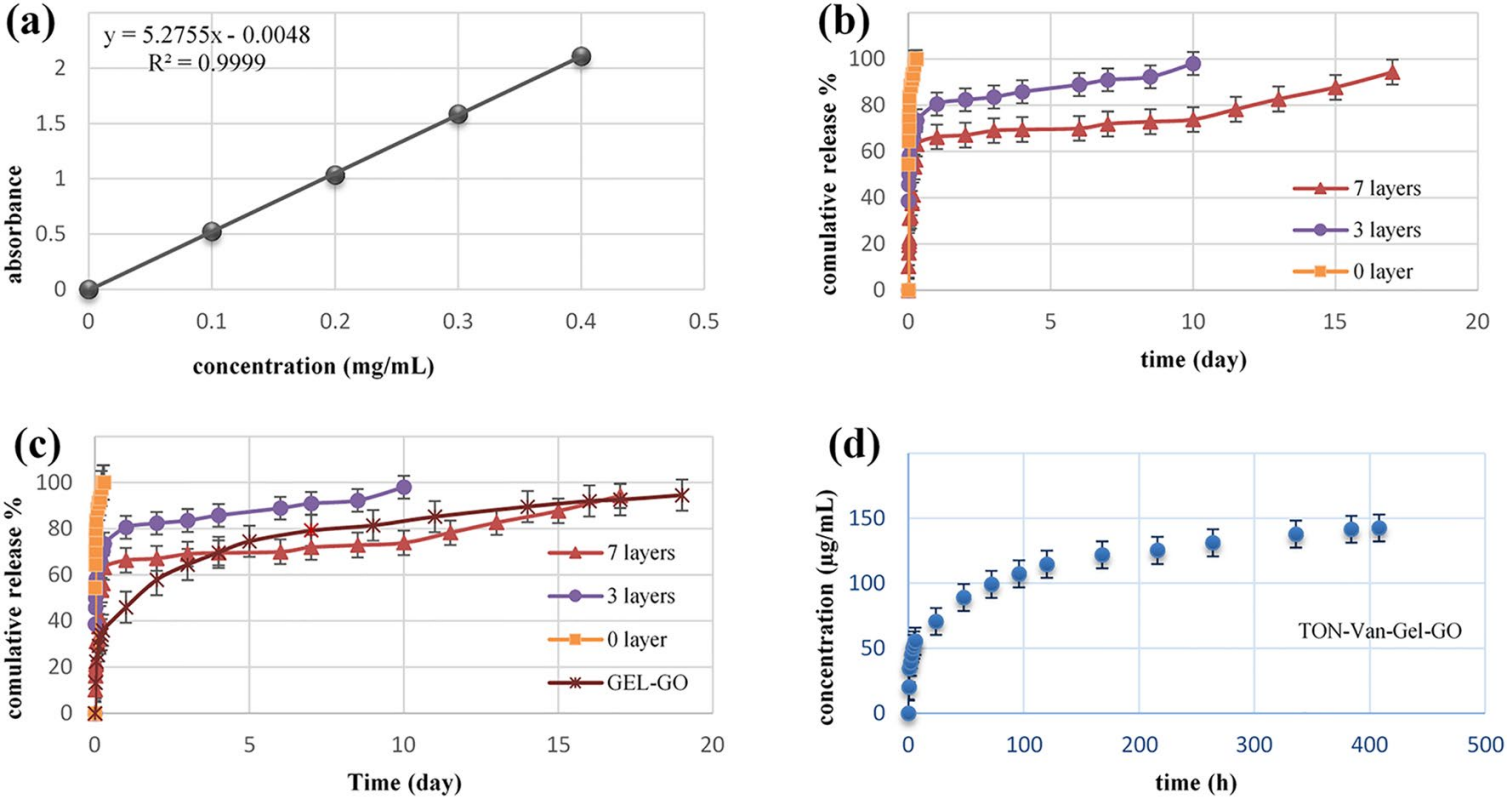
**(a)** Calibration curve of serial dilutions of Vancomycin in PBS, **(b**) Vancomycin release profile from different layers of gelatin coating, **(c)** Vancomycin release profile from gelatin-GO coating and **(d)** concentration of released drug from TON-Gel-GO.

The effect of gelatin thickness on the release of Vancomycin was investigated from TON-Van, TON-Van-Gel C3, and TON-Van-Gel C7. The thickness of 3 and 7 layers of gelatin was 445 nm and 1038.06 nm, obtained by ellipsometry. As it can be seen in Fig. 6a,b, the ellipsometry results were confirmed by the SEM cross sectional images. Figure 7a shows the Vancomycin calibration curve (in PBS, at 280 nm) that used to obtain Vancomycin concentrations. The total amount of Vancomycin loaded into titania nanotubes was measured 490 µg/cm^2^ using a UV–Vis spectrophotometer. Figure 7b presents a comparative drug release profile of Vancomycin from TON-Van, TON-Van-Gel C3, and C7. As anticipated, the release behavior of Vancomycin from all the samples displayed biphasic behavior, including a burst release and sustained release. Drug molecules in the upper part of the nanotubes were released immediately in the media at the initial stage (burst release phase), inhibiting the bacterial invasion and improve the antibacterial efficacy in early hours of implantation. Drug release profile of the samples are provided in Table 4. In the burst release phase, the Vancomycin released from TON-Van was approximately 83% in the first hour whereas the released drug from TON-Van-Gel C3 and C7 were 58% and 31%, respectively. In the sustained release phase, the drug was released from TON-Van, TON-Van-Gel C3, and C7 in 1, 10, and 17 days, respectively. The slower release of Vancomycin from TON-Van in the second stage demonstrates that the nanotube structure could act as a nano-reservoir for the drug. In addition, the results show that the gelatin-coating reduced the amount of released drug in the burst release phase. With increasing the number of layers of spin-coated gelatin, the thickness would increase which resulted in the significant decrease in the rate of drug release. This is probably because the polymer chains could restrict the movement of drug molecules and limits their release. The limitation in the rate of drug release would extend the time which provides more control on drug release profile that enhances the antibacterial property of the samples.

**Table 4:** Vancomycin release profile from different samples.

Sample	% Drug release			Drug totally released (day)
	1 h	1 day	7 days	
TON-Van	83.47	100	100	1
TON-van-Gel C3	58.28	80.56	90.98	10 ± 1
TON-van-Gel C7	31.13	66.28	71.92	17 ± 1
TON-van-Gel-GO	22.34	45.9	79.17	19 ± 1

The effect of graphene oxide on the release profile of the Vancomycin was investigated by TON-Van-Gel-GO. As it can be seen in Fig. 7c, a biphasic behavior for GO-gelatin sample was detect. The percentage of drug release was decreased from 31 to 22% in the burst release stage by adding the GO nanoparticles (Table 4). The negative charge of graphene oxide and carboxyl groups could electrostatically interact with chains of gelatin. Hence, the physical links between gelatin chains create difficulty for Vancomycin molecules to get released into PBS which results in prolonged release period. In fact, the GO-gelatin coating could prolong Vancomycin release up to 19 days. In comparison, the GO-gelatin coated samples could provide longer and sustained drug release required for successful anti-infection bone therapy as compared to TON-Van and TON-Van-Gel C7 samples.

In order to have an effective local drug delivery system, the concentration of antibacterial drug release in the media should be blow the toxic level and above the minimum inhibitory concentration (MIC). Here, Vancomycin did not show toxic behavior at 800 µg/mL for MG-63 Cells. Moreover, Vancomycin has been reported to have a MIC of 0.5–10 µg/mL for *S*. *aureus*^55–59^. As shown in Fig. 7d, the Vancomycin concentration for TON-Van-Gel-GO in the media was within the therapeutic window. This could mean that the drug delivery system should be able to kill the bacteria and avoid the formation of biofilm without interference with the cellular process.

Mathematical modeling was used to investigate the mechanism of drug release and analyze the release kinetics. Model-dependent methods based on different mathematical functions were employed to select the best fit for Vancomycin release profile with a higher value. Table 5 summarizes the equation of mathematical functions used to fit the release experiments data including zero-order, first-order, Higuchi, and Hixson-Crowell. The obtained ${\text{R}}^{2}$ values for the samples (TON-Van, TON-Van-Gel C7, and TON-Van-Gel-GO) are listed in Table 6. The comparison between the ${\text{R}}^{2}$ values revealed that the Vancomycin release was perfectly fitted with the first-order model in all the samples. This is an indication for water-soluble drug release from a porous matrix or a matrix with diffusion-controlled release system.

**Table 5:** The mathematical function equation used to fit the release data.

Zero order	$Q_{{\text{t}}} = Q_{0} + k_{0} t$
First order	$\ln Q_{{\text{t}}} = Q_{0} - k_{1} t$
Higuchi	$Q_{{\text{t}}} = k_{{\text{H}}} t^{0.5}$
Hixson-Crowell	$Q_{0}^{1/3} - Q_{{\text{t}}}^{1/3} = K_{{{\text{hc}}}} t$

**Table 6:** ${\text{R}}^{2}$ values of Vancomycin-delivery kinetics.

Kinetic model	Samples	First order	Zero order	Hixson-Crowell	Higuchi
${\text{R}}^{2}$	TON-Van-Gel C7	0.8346	0.7097	0.8239	0.8053
${\text{R}}^{2}$	TON-Van-Gel-GO	0.9703	0.8115	0.9281	0.9561
${\text{R}}^{2}$	TON-Van	0.9647	0.7333	0.911	0.8711

### Cell viability

**Figure 8:**
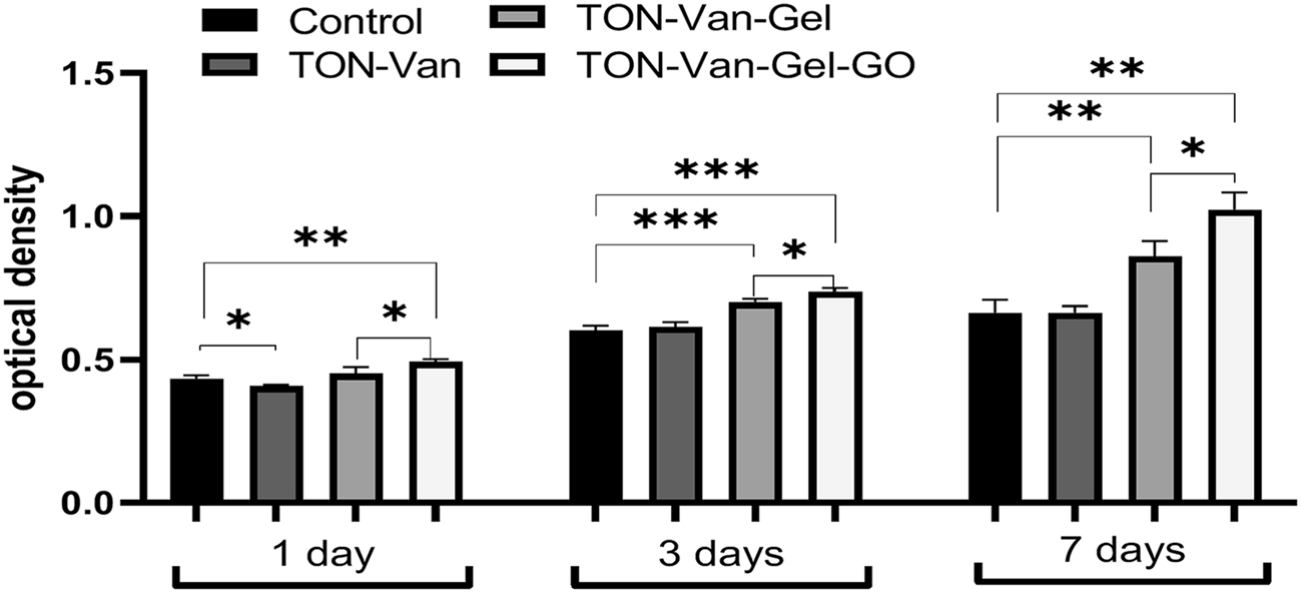
Cell viability of MG-63 cells cultured on TON-Van, TON-Van-Gel, TON-Van-Gel-GO at different time point using MTT assay. (*p < 0.05, **p < 0.01, ***p < 0.001, n = 3).

The cell viability and proliferation on the surface of the samples were evaluated by MTT assay. Figure 8 shows MG-63 cell proliferation after 1, 3, and 7 days of culture. As it can be seen, no statistically significant difference between TON without coating and control sample was observed. Moreover, the number of viable cells on the surface of TON-Van was significantly lower than the control after the day 1. The gelatin-based coating showed to remarkably improve cell viability and the addition of GO nanoparticles had a beneficial impact on cell viability of the samples. It is known gelatin could provide cells with the biomimetic bone environment since it is the major protein of the extracellular matrix. Gelatin has the arginine–glycine–aspartic acid (RGD) sequence which is known for establishing cell-substrate interactions. Moreover, the dispersion of GO nanoparticles into gelatin increases the chance to create more cellular niches for osteoblast cells due to the enlargement of the surface area and the increase of surface roughness. It is known that the surface oxygen-containing functional groups trigger the adsorption of the surrounding serum proteins on the surface. This could be the reason that GO-enriched surfaces would absorb the exogenous proteins that results in eliciting efficient interactions with the cells^51^. It is shown that the presence of GO nanoparticles could engage in gene expression profile and upregulate mRNA expression levels of all osteogenic markers^60,61^.

In addition, GO nanosheets could interfere in apatite nucleation and hydroxyl apatite (HA) formation. It is shown by Wang et al. this could be originated from ion interactions. The –OH and carboxyl groups from GO could attract Ca^2+^ and HPO_4_^2-^ of microenviroment solution resulting in the accelerated hydroxy apatite formation^50^.

The TON-Van-Gel-GO sample showed the best cell substrate interactions with MG-63 cells. Here, Vancomycin at 800 µg/mL showed about 80% cell viability at day 1. Therefore, the results show no cytotoxicity whether by the samples or the dosage of the drug used according to ISO-10993-5.

### Bioactivity

**Figure 9:**
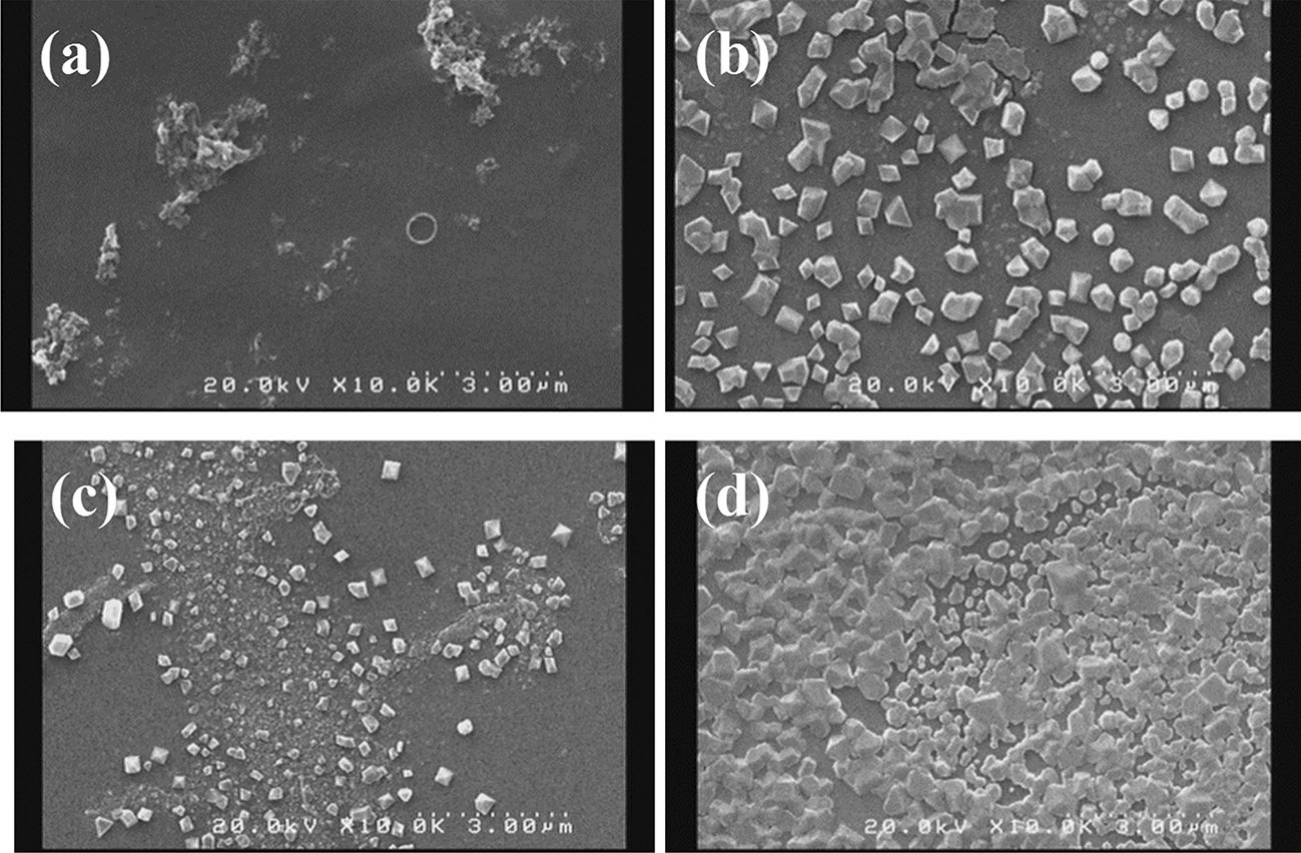
SEM images of TON coated by gelatin with and without GO soaking in SBF solution for different times **(a)** TON-Gel for 3 days **(b)** TON-Gel-GO for 3 days, **(c)** TON-Gel for 7 days, and **(d)** TON-Gel-GO for 7 days.

Previous studies have demonstrated that both pure titanium and TON surfaces are bio-inert^62–64^. FESEM was utilized to determine the formation of bone-like apatite on the samples (Gelatin and Gelatin-GO) soaked in SBF solution for 3 and 7 days. As shown in Fig. 9a, a few apatite nucleates were formed on the surface of gelatin coated sample after 3 days whereas GO-gelatin coated sample was covered by flake-like apatite (Fig. 9b). The negative charge of GO with deprotonation of –COOH and –OH groups could attract ${\text{Ca}}^{2 + }$ ions and enhance the apatite nucleation^50^. After 7 days of immersion in SBF, the amount of apatite crystals on both samples were increased (Fig. 9c,d). However, the mineralization on the surface of GO reinforced sample was higher than the sample without GO, suggesting that the sample coated by gelatin and reinforced with GO has the highest bioactivity among the samples. The amino groups in gelatin can attract calcium ions followed by ${\text{PO}}_{4}^{3 - }$ which could results in accelerated biomineralization process. GO also promotes the flake-like hydroxyapatite deposition through electrostatic interaction. It is fair to assume that TON-Van-Gel-GO sample, could provide a more stable connection between the surface of samples and bone tissues.

### The antibacterial assay

**Figure 10:**
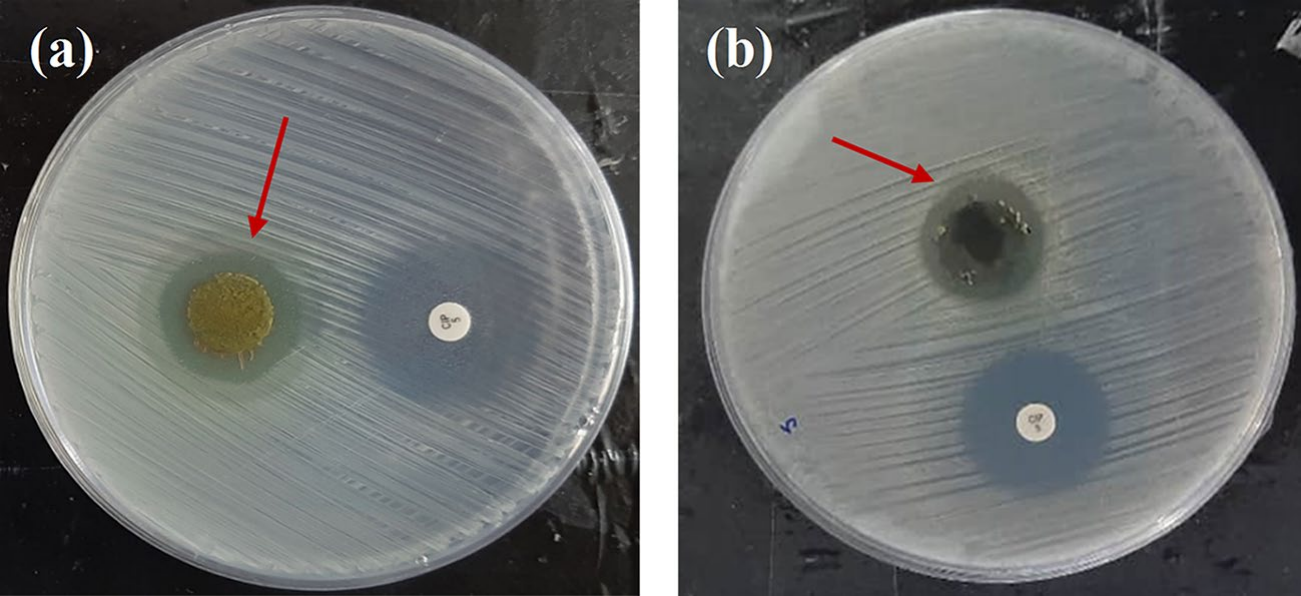
Disc diffusion assay to evaluate the antibacterial property of **(a)** TON-Van-Gel C7, and **(b)** TON-Van-Gel-GO.

The antibacterial characteristic of the samples was investigated by disc diffusion assay after 24 h of incubation against gram-positive *S*. *aureus* bacteria, the most common pathogenic bacteria in bone infections. Figure 10, shows the inhibition zones of the samples including TON-Van-Gel and TON-Van-Gel-GO. The inhibition zones diameters were also measured and depicted in Table 7 which confirm our observation. The inhibition zones of both samples were about 20 mm which indicates both samples provide Vancomycin delivery models that can inhibit *S*. *aureus* infection. It is apparent that Vancomycin could provide an effective antibacterial property against gram-positive bacteria such as *S*. *aureus* with minimal cytotoxicity. This data was in agreement with Liu et al.^65^ finding which confirmed that Vancomycin sustained delivery from titanium surface mediated by nanoparticles provided the similar inhibition zone and showed efficient antibacterial effect against *S. aureus*. The proper pattern of drug release is essential for the effective antibacterial ability of the implant`s surface. As discussed in 3.6, in this study, the concentration of released vancomycin from coating reached the concentration above the MIC in the first hour when the possibility of infection is higher. Also, based on Zhang et al.^66^ study which developed an electrospun Vancomycin-loaded coating on titanium substrate, the obtained profile of release and the concentration of the released Vancomycin during 19 days may support treating implant-associated infection in-vivo.

**Table 7:** The values ± SD for inhibition zone obtained from disc diffusion assay against *S*. *aureus*.

Samples	Control	TON-Van-Gel C7	TON-Van-Gel-GO
Zone of inhibition (mm)	25 ± 1.1	20 ± 1.6	20 ± 1.3

## Conclusion

In the present study, a layer of ${\text{TiO}}_{2}$ nanotubes on pure titanium samples was fabricated through electrochemical anodization in order to carry the vancomycin, as an antibacterial agent. A gelatin-based coating which was reinforced with graphene oxide was spin-coated on the surface of the samples to control the release profile while improve the biological activity of the samples. The abundant formation of apatite crystals on the Gelatin-GO substrate implies that TON-Van-Gel-GO has the superior bioactivity and possible improved osteointegration. Since the TON-Van-Gel-GO also showed effective antibacterial properties against gram-positive *S*. *aureus*, it is believed that it could present a promising potential for bone tissue engineering applications.

## Data Availability

The datasets used and/or analyzed during the current study available from the corresponding author on reasonable request.
